# Crystal structure of (1*R*,4*R*)-*tert*-butyl 3-oxo-2-oxa-5-aza­bicyclo­[2.2.2]octane-5-carboxyl­ate

**DOI:** 10.1107/S2056989015010476

**Published:** 2015-06-06

**Authors:** Suvratha Krishnamurthy, Venkataprasad Jalli, Tarun Chand Vagvala, Tetsuji Moriguchi, Akihiko Tsuge

**Affiliations:** a Department of Applied Chemistry, Kyushu Institute of Technology, Kitakyushu 804-8550, Japan; b Graduate School of Life Sciences and Systems Engineering, Kyushu Institute of Technology, Kitakyushu 804-8550, Japan

**Keywords:** crystal structure, (1*R*,4*R*)- aza-oxa bicyclic chiral lactone

## Abstract

In the title compound, C_11_H_17_NO_4_, commonly known as *N*-*tert*-but­oxy­carbonyl-5-hy­droxy-d-pipecolic acid lactone, the absolute configuration is (1*R*,4*R*) due to the enantiomeric purity of the starting material which remains unchanged during the course of the reaction. In the crystal there no inter­molecular hydrogen bonds.

## Related literature   

For background information on 5-hy­droxy­pipecolic acid and related compounds, see: Witkop & Foltz (1957 ▸); Hoarau *et al.* (1996 ▸); Sun *et al.* (2008 ▸). For the synthesis of a related compound, see: Krishnamurthy *et al.* (2014 ▸). For crystal structures of related lactones, see: (1*S*,4*S*) conformer, racemic mixture, Moriguchi, Krishnamurthy, Arai & Tsuge (2014 ▸); Moriguchi, Krishnamurthy, Arai, Matsumoto *et al.* (2014 ▸). 
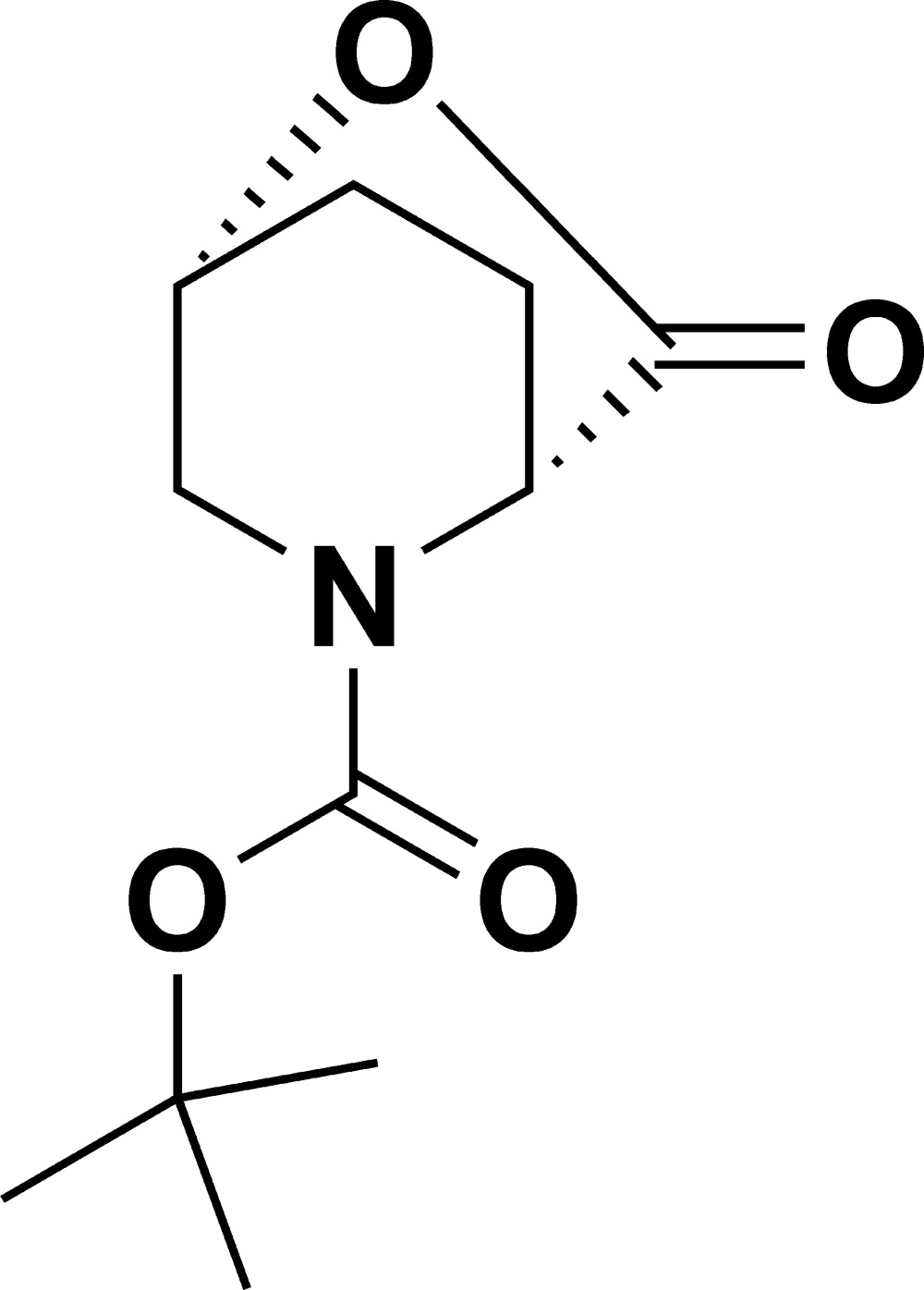


## Experimental   

### Crystal data  

C_11_H_17_NO_4_
*M* *_r_* = 227.26
Orthorhombic, 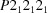

*a* = 9.6472 (4) Å
*b* = 9.7084 (4) Å
*c* = 12.2323 (5) Å
*V* = 1145.66 (8) Å^3^
*Z* = 4
Mo *K*α radiation
μ = 0.10 mm^−1^
*T* = 90 K
0.45 × 0.40 × 0.40 mm

### Data collection  

Bruker APEX2 KY CCD diffractometer
Absorption correction: multi-scan *SADABS* (Bruker, 2009 ▸) *T*_min_ = 0.870, *T*_max_ = 0.961
13518 measured reflections
2791 independent reflections
2728 reflections with *I* > 2σ(*I*)
*R*_int_ = 0.021

### Refinement  

*R*[*F* ^2^ > 2σ(*F* ^2^)] = 0.030
*wR*(*F* ^2^) = 0.081
*S* = 1.03
2791 reflections
148 parameters
H-atom parameters constrained
Δρ_max_ = 0.24 e Å^−3^
Δρ_min_ = −0.27 e Å^−3^
Absolute structure: Flack (1983 ▸), 2933 Friedel pairs
Absolute structure parameter: 0.1 (7)

### 

Data collection: *APEX2* (Bruker,2009 ▸); cell refinement: *SAINT* (Bruker, 2009 ▸); data reduction: *SAINT*; program(s) used to solve structure: *SHELXS97* (Sheldrick, 2008 ▸); program(s) used to refine structure: *SHELXL97* (Sheldrick, 2008 ▸); molecular graphics: *SHELXTL* (Sheldrick, 2008 ▸); software used to prepare material for publication: *SHELXL97*.

## Supplementary Material

Crystal structure: contains datablock(s) global, I. DOI: 10.1107/S2056989015010476/zs2333sup1.cif

Structure factors: contains datablock(s) I. DOI: 10.1107/S2056989015010476/zs2333Isup2.hkl

Supporting information file. DOI: 10.1107/S2056989015010476/zs2333Isup3.cml

**Fig. 1.**
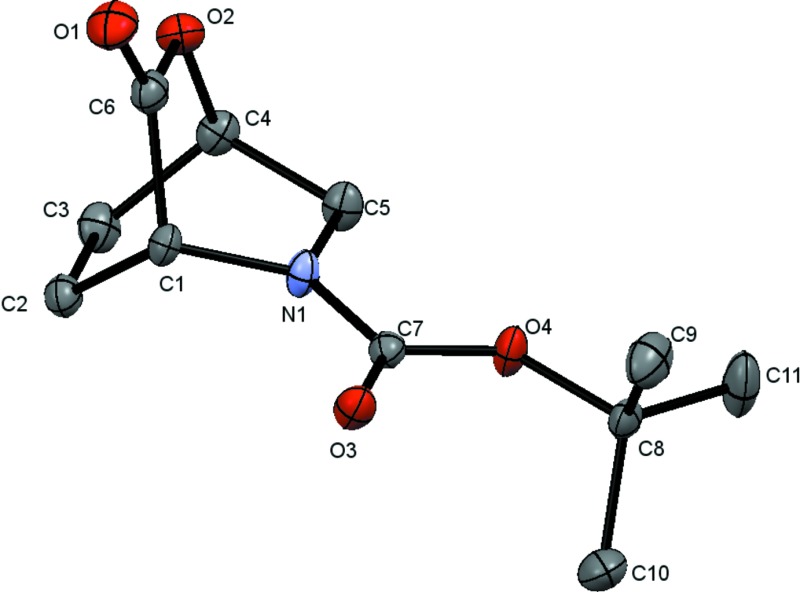
Molecular configuration and atom numbering scheme for the title compound with displacement ellipsoids drawn at the 50% probability level. Hydrogen atoms are omitted for clarity.

. DOI: 10.1107/S2056989015010476/zs2333fig1.tif
Mol­ecular configuration and atom numbering scheme for the title compound with displacement ellipsoids drawn at the 50% probability level. Hydrogen atoms are omitted for clarity.

**Fig. 2.**
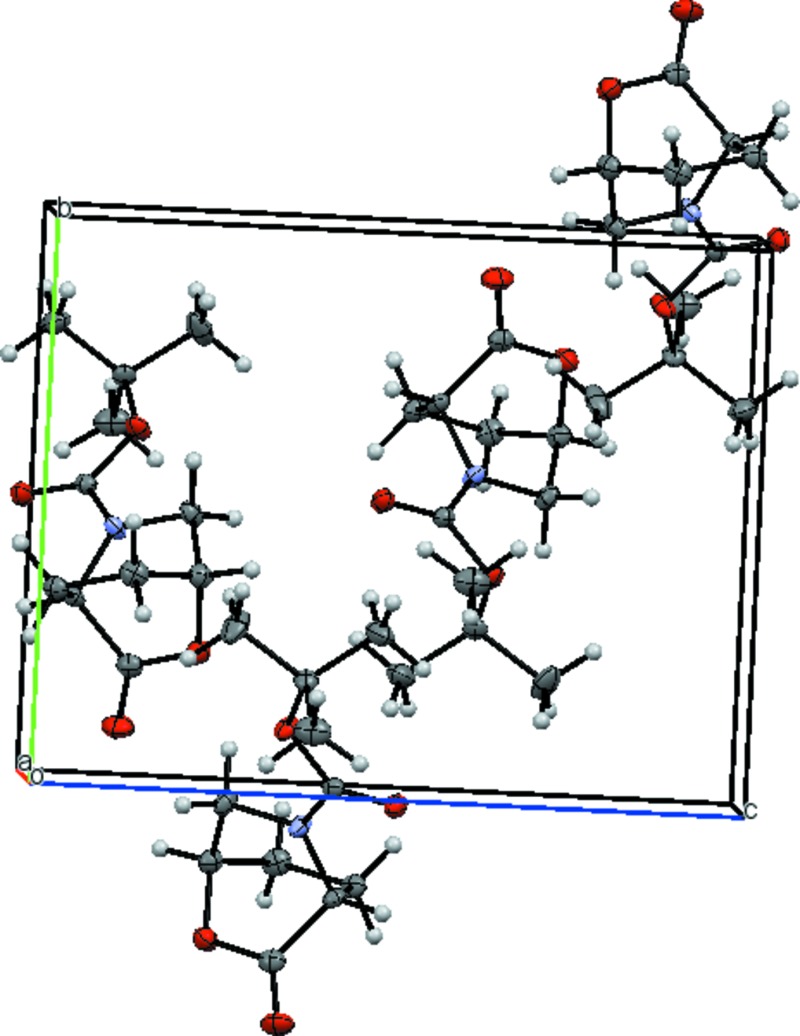
Crystal packing diagram of the title compound.

. DOI: 10.1107/S2056989015010476/zs2333fig2.tif
Crystal packing diagram of the title compound.

**Fig. 3.**
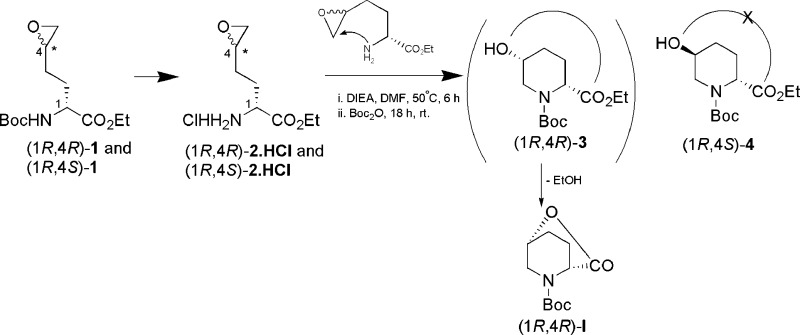
Synthetic scheme for the title compound (I).

. DOI: 10.1107/S2056989015010476/zs2333fig3.tif
Synthetic scheme for the title compound (I).

CCDC reference: 1062075

Additional supporting information:  crystallographic information; 3D view; checkCIF report

## supplementary crystallographic information

### S1. Comment

5-Hydroxypipecolic acid is a higher homologue of 4-hydroxyproline, which is found in dates (Witkop & Foltz, 1957). 4-Hydroxyproline is formed by post-translational modification of proline in collagen and is responsible for enhancing its stability. Literature reports the synthesis of 5-hydroxypipecolic acid derivatives (Hoarau *et al.*, 1996; Sun *et al.*, 2008), generally forming diastereomeric mixtures of *cis*- and *trans*-5-hydroxypipecolic acids. Therefore such syntheses suffer from disadvantages of separation of the diasteromers making the procedure very tedious. A facile procedure to isolate this amino acid was desirable.

Our previous communication reported the synthesis of a 4-hydroxyproline derivative from an amino acid bearing epoxide (Krishnamurthy *et al.* 2014). It is reported in this study that the *cis*-isomer undergoes intramolecular lactonization to *tert*-butyl- 3-oxo-2-oxa-5-azabicyclo[2.2.1]heptane-5-carboxylate, making the isolation from the *trans* ester highly feasible. Based on this observation it can be expected that *cis*-5-hydroxypipecolic acids would also undergo *in situ* intramolecular lactonization. In fact, when a mixture of a *cis*- and *trans*-5-hydroxypipecolic acid derivatives was reacted under acidic conditions, the *cis*-isomer successfully converted to the lactone (I), subsequently readily separated from the remaining *trans-*-isomer. We had previously reported the crystal structure of racemic *tert*-butyl- 3-oxo-2-oxa-5-azabicyclo[2.2.2]octane-5-carboxylate (Moriguchi, Krishnamurthy, Arai, Tsuge *et al.*, 2014) and (1*S*,4*S*)-*tert*-butyl 3-oxo-2-oxa-5-azabicyclo[2.2.2]octane-5-carboxylate (Moriguchi, Krishnamurthy, Arai, & Tsuge, 2014). Herein we would like to report the crystal structure of enantiomerically pure (1*R*,4*R*)-*tert*-butyl- 3-oxo-2-oxa-5-azabicyclo[2.2.2]octane-5-carboxylate, C_11_H_17_N O_4_, (I).

The title compound (I), commonly known as *N*-*tert*- butoxycarbonyl-5-hydroxy-D-pipecolic acid lactone, was derived from a starting product having a *cis* configuration for both hydroxyl and carboxyl groups, leading to lactone formation (Fig. 1). The nitrogen atom N1 appears next to the bridge-head atom within the bicyclic ring system. The absolute configuration of the compound was found to be (1*R*,4*R*) due to the configuration of the starting material (Fig. 3). The Flack structure parameter (Flack, 1983) determined for (I) [0.1 (7)], although not definitive because of the uncertainty factor, is considered to provide adequate supporting evidence for this configuration. The desired hydrophobic conformer (1*R*,4*R*), (I) was easily isolated from hydrophylic (1*R*,4*S*)–(4) (Fig. 3). The intramolecular lactonization is possible only in (1*R*,4*R*)–(3) isomer due to its configuration. The hydroxyl and the carboxyl groups are in close proximity due to the *cis*- configuration of (1*R*,4*R*)–(3), which leads to the intamolecular lactonization with loss of EtOH. With the (1*R*,4*S*)–(4) isomer the hydroxyl and the carboxyl groups are far apart due to the *trans*- configuration, thus preventing the lactonization. In the crystal there no formal intramolecular hydrogen bonds (Fig. 2).

This work represents the first structural characterization of this (1*R*,4*R*)- aza and oxa bicyclic chiral lactone characterized by X-ray analysis.

### S2. Experimental

The basic reaction scheme for preparation of the title compound (I) is shown in Fig. 3. To a ice cooled solution of 4 mol/L HCl in 1,4-dioxane (16 mL), a solution of diastereomeric (1) ( 0.97 g, 3.56 mmol) in 0.5 mL of 1,4-dioxane was added. This reaction mixture was then warmed to room temperature and stirred. After 3 h most of the volatile materials were removed under vacuum resulting in a crude oily mixture. Trituration with diethyl ether followed by decantation resulted in (2) as a foam (0.71 g, 95 %). DIEA (0.89 mL, 5.07 mmol) was added to a solution of (2) ( 0.71 g, 3.38 mmol) in DMF (13 mL) and stirred at 50 °C. After 6 h the solution was warmed to room temperature, followed by addition of Boc_2_O (3.96 g, 18.1 mmol), additional DIEA (0.3 mL, 1.69 mmol) and stirred at room temperature for 18 h. The DMF was evaporated and the crude mixture was subsequently washed with 10% aqueous citric acid, 4% aqueous NaHCO_3_, brine, dried (MgSO_4_), filtered and evaporated to obtain an oil. The crude product was purified by silica gel column chromatography with (CHCl_3_/MeOH, 100:0 to 98:2, *v/v*) to yield (1*R*,4*R*), (I) (0.18 g, 23%) as a white solid. Single crystals were obtained by vapour diffusion method at room temperature, i.e., hexane vapour was allowed to diffuse into an EtOAc (0.5 ml) solution of (1*R*,4*R*), (I) at room temperature. Single crystals suitable for analysis were obtained after a week.

^1^H NMR 4.61-4.82 (2H, m), 3.63 (1H, m), 3.45 (1H, m), 2.22 (1H, br s), 2.11 (1H, m), 2.00 (1H, m), 1.80 (1H, m), 1.47 (9H, s); MS (FAB *m/z*): 228 (74), 190 (44), 172 (100), 137 (50), 128 (68), 55 (47). HRMS(FAB) calcd for C_11_H_18_N_1_O_4_ [M + H]^+^ 228.12358, found 228.1243

### S3. Refinement

All hydrogen atoms were placed in calculated positions (C—H = 0.98–1.00 Å) and allowed to ride, with *U*_iso_H = 1.5*U*_eq_C(methyl) or 1.2*U*_eq_C(methine and methylene). The absolute structure parameter (Flack, 1983) for (I) [0.01 (7) for 2933 Friedel pairs], although not definitive is sufficient to confirm the (1*R*,4*R*) identity, as distinct from that of the known (1*S*,4*S*) conformer (Moriguchi, Krishnamurthy, Arai & Tsuge, 2014).

### Crystal data

**Table:**

C_11_H_17_NO_4_	*D*_x_ = 1.318 Mg m^−^^3^
*M**_r_* = 227.26	Mo *K*α radiation, λ = 0.71073 Å
Orthorhombic, *P*2_1_2_1_2_1_	Cell parameters from 9630 reflections
*a* = 9.6472 (4) Å	θ = 2.7–28.7°
*b* = 9.7084 (4) Å	µ = 0.10 mm^−^^1^
*c* = 12.2323 (5) Å	*T* = 90 K
*V* = 1145.66 (8) Å^3^	Prism, colorless
*Z* = 4	0.45 × 0.40 × 0.40 mm
*F*(000) = 488	

### Data collection

**Table:**

Bruker APEX2 KY CCD diffractometer	2791 independent reflections
Radiation source: fine-focus sealed tube	2728 reflections with *I* > 2σ(*I*)
Graphite monochromator	*R*_int_ = 0.021
Detector resolution: 16.6666 pixels mm^-1^	θ_max_ = 28.7°, θ_min_ = 2.7°
φ and ω–scans	*h* = −12→12
Absorption correction: multi-scan *SADABS* (Bruker, 2009)	*k* = −12→12
*T*_min_ = 0.870, *T*_max_ = 0.961	*l* = −16→16
13518 measured reflections	

### Refinement

**Table:**

Refinement on *F*^2^	Hydrogen site location: inferred from neighbouring sites
Least-squares matrix: full	H-atom parameters constrained
*R*[*F*^2^ > 2σ(*F*^2^)] = 0.030	*w* = 1/[σ^2^(*F*_o_^2^) + (0.0583*P*)^2^ + 0.1302*P*] where *P* = (*F*_o_^2^ + 2*F*_c_^2^)/3
*wR*(*F*^2^) = 0.081	(Δ/σ)_max_ < 0.001
*S* = 1.02	Δρ_max_ = 0.24 e Å^−^^3^
2791 reflections	Δρ_min_ = −0.27 e Å^−^^3^
148 parameters	Extinction correction: *SHELXL97*
0 restraints	Extinction coefficient: 0.0015
Primary atom site location: structure-invariant direct methods	Absolute structure: Flack (1983), 2933 Friedel pairs
Secondary atom site location: difference Fourier map	Absolute structure parameter: 0.1 (7)

### Special details

**Table:**

Geometry. All e.s.d.'s (except the e.s.d. in the dihedral angle between two l.s. planes) are estimated using the full covariance matrix. The cell e.s.d.'s are taken into account individually in the estimation of e.s.d.'s in distances, angles and torsion angles; correlations between e.s.d.'s in cell parameters are only used when they are defined by crystal symmetry. An approximate (isotropic) treatment of cell e.s.d.'s is used for estimating e.s.d.'s involving l.s. planes.
Refinement. Refinement of *F*^2^ against ALL reflections. The weighted *R*-factor *wR* and goodness of fit *S* are based on *F*^2^, conventional *R*-factors *R* are based on *F*, with *F* set to zero for negative *F*^2^. The threshold expression of *F*^2^ > σ(*F*^2^) is used only for calculating *R*-factors(gt) *etc*. and is not relevant to the choice of reflections for refinement. *R*-factors based on *F*^2^ are statistically about twice as large as those based on *F*, and *R*- factors based on ALL data will be even larger.

### Fractional atomic coordinates and isotropic or equivalent isotropic displacement parameters (Å^2^)

**Table:**

	*x*	*y*	*z*	*U*_iso_*/*U*_eq_	
C1	0.65404 (10)	0.31574 (9)	0.06026 (7)	0.01427 (18)	
H1	0.71	0.2937	−0.0063	0.017*	
C2	0.49996 (10)	0.33409 (10)	0.03171 (8)	0.0176 (2)	
H2A	0.4628	0.2485	−0.0011	0.021*	
H2B	0.4883	0.41	−0.0215	0.021*	
C3	0.42204 (10)	0.36786 (11)	0.13907 (9)	0.0206 (2)	
H3B	0.378	0.4597	0.1335	0.025*	
H3A	0.3485	0.2987	0.1521	0.025*	
C4	0.52501 (11)	0.36654 (10)	0.23335 (8)	0.01785 (19)	
H4	0.475	0.3836	0.3037	0.021*	
C5	0.63878 (10)	0.47291 (11)	0.21909 (8)	0.0178 (2)	
H5B	0.5993	0.567	0.2175	0.021*	
H5A	0.7064	0.4671	0.2798	0.021*	
C6	0.66134 (10)	0.19956 (10)	0.14366 (8)	0.01762 (19)	
C7	0.81399 (10)	0.51222 (9)	0.07261 (8)	0.01444 (19)	
C8	0.96933 (10)	0.70326 (10)	0.12123 (8)	0.01618 (19)	
C9	1.10001 (11)	0.61760 (12)	0.11084 (11)	0.0276 (2)	
H9A	1.0964	0.5638	0.0431	0.041*	
H9B	1.1072	0.5551	0.1735	0.041*	
H9C	1.181	0.6785	0.1092	0.041*	
C10	0.94288 (14)	0.79172 (11)	0.02135 (10)	0.0272 (3)	
H10A	0.8622	0.8507	0.0345	0.041*	
H10B	0.925	0.7324	−0.0419	0.041*	
H10C	1.0243	0.8492	0.0069	0.041*	
C11	0.97332 (14)	0.79157 (13)	0.22359 (10)	0.0311 (3)	
H11A	0.9866	0.7325	0.2877	0.047*	
H11B	0.8858	0.842	0.2307	0.047*	
H11C	1.0502	0.8572	0.2184	0.047*	
N1	0.70560 (9)	0.44009 (8)	0.11448 (7)	0.01612 (17)	
O1	0.71703 (8)	0.08995 (8)	0.13238 (7)	0.02526 (18)	
O2	0.59161 (8)	0.23044 (8)	0.23684 (6)	0.01975 (16)	
O3	0.86922 (8)	0.48744 (7)	−0.01469 (6)	0.01893 (16)	
O4	0.84801 (7)	0.61426 (7)	0.14236 (6)	0.01846 (16)	

### Atomic displacement parameters (Å^2^)

**Table:**

	*U*^11^	*U*^22^	*U*^33^	*U*^12^	*U*^13^	*U*^23^
C1	0.0141 (4)	0.0119 (4)	0.0168 (4)	−0.0019 (3)	0.0008 (3)	−0.0028 (3)
C2	0.0155 (5)	0.0172 (4)	0.0201 (4)	−0.0010 (4)	−0.0030 (3)	−0.0007 (3)
C3	0.0129 (4)	0.0230 (5)	0.0259 (5)	0.0005 (4)	0.0000 (4)	−0.0014 (4)
C4	0.0160 (4)	0.0179 (4)	0.0196 (4)	−0.0001 (4)	0.0032 (4)	−0.0012 (4)
C5	0.0166 (4)	0.0198 (4)	0.0172 (4)	−0.0019 (4)	0.0050 (3)	−0.0052 (3)
C6	0.0143 (4)	0.0171 (4)	0.0215 (5)	−0.0016 (3)	−0.0017 (4)	−0.0002 (4)
C7	0.0145 (4)	0.0126 (4)	0.0162 (4)	0.0002 (3)	−0.0017 (3)	0.0000 (3)
C8	0.0151 (4)	0.0155 (4)	0.0179 (4)	−0.0058 (4)	−0.0001 (3)	−0.0001 (3)
C9	0.0166 (5)	0.0241 (5)	0.0421 (6)	−0.0010 (4)	−0.0024 (4)	0.0012 (5)
C10	0.0361 (6)	0.0179 (5)	0.0275 (5)	−0.0039 (4)	−0.0051 (4)	0.0051 (4)
C11	0.0330 (6)	0.0349 (6)	0.0255 (6)	−0.0171 (5)	0.0039 (5)	−0.0126 (5)
N1	0.0161 (4)	0.0161 (4)	0.0162 (4)	−0.0040 (3)	0.0032 (3)	−0.0056 (3)
O1	0.0252 (4)	0.0171 (3)	0.0334 (4)	0.0050 (3)	−0.0025 (4)	0.0002 (3)
O2	0.0211 (4)	0.0186 (3)	0.0195 (3)	0.0009 (3)	0.0015 (3)	0.0029 (3)
O3	0.0219 (4)	0.0184 (3)	0.0165 (3)	−0.0038 (3)	0.0041 (3)	−0.0022 (3)
O4	0.0175 (3)	0.0185 (3)	0.0194 (3)	−0.0073 (3)	0.0040 (3)	−0.0059 (3)

### Geometric parameters (Å, º)

**Table:**

C1—N1	1.4645 (11)	C6—O2	1.3570 (12)
C1—C6	1.5225 (13)	C7—O3	1.2175 (12)
C1—C2	1.5373 (14)	C7—O4	1.3481 (11)
C1—H1	1.0	C7—N1	1.3587 (12)
C2—C3	1.5482 (14)	C8—O4	1.4776 (11)
C2—H2A	0.99	C8—C9	1.5156 (15)
C2—H2B	0.99	C8—C10	1.5150 (14)
C3—C4	1.5222 (15)	C8—C11	1.5180 (14)
C3—H3B	0.99	C9—H9A	0.98
C3—H3A	0.99	C9—H9B	0.98
C4—O2	1.4699 (13)	C9—H9C	0.98
C4—C5	1.5171 (13)	C10—H10A	0.98
C4—H4	1.0	C10—H10B	0.98
C5—N1	1.4677 (12)	C10—H10C	0.98
C5—H5B	0.99	C11—H11A	0.98
C5—H5A	0.99	C11—H11B	0.98
C6—O1	1.2000 (12)	C11—H11C	0.98
			
N1—C1—C6	106.95 (7)	O3—C7—O4	126.43 (9)
N1—C1—C2	109.62 (8)	O3—C7—N1	124.45 (9)
C6—C1—C2	106.43 (8)	O4—C7—N1	109.12 (8)
N1—C1—H1	111.2	O4—C8—C9	110.65 (8)
C6—C1—H1	111.2	O4—C8—C10	109.82 (8)
C2—C1—H1	111.2	C9—C8—C10	112.55 (9)
C1—C2—C3	107.54 (8)	O4—C8—C11	101.89 (8)
C1—C2—H2A	110.2	C9—C8—C11	110.98 (10)
C3—C2—H2A	110.2	C10—C8—C11	110.44 (9)
C1—C2—H2B	110.2	C8—C9—H9A	109.5
C3—C2—H2B	110.2	C8—C9—H9B	109.5
H2A—C2—H2B	108.5	H9A—C9—H9B	109.5
C4—C3—C2	108.91 (8)	C8—C9—H9C	109.5
C4—C3—H3B	109.9	H9A—C9—H9C	109.5
C2—C3—H3B	109.9	H9B—C9—H9C	109.5
C4—C3—H3A	109.9	C8—C10—H10A	109.5
C2—C3—H3A	109.9	C8—C10—H10B	109.5
H3B—C3—H3A	108.3	H10A—C10—H10B	109.5
O2—C4—C5	107.40 (8)	C8—C10—H10C	109.5
O2—C4—C3	108.35 (8)	H10A—C10—H10C	109.5
C5—C4—C3	112.29 (9)	H10B—C10—H10C	109.5
O2—C4—H4	109.6	C8—C11—H11A	109.5
C5—C4—H4	109.6	C8—C11—H11B	109.5
C3—C4—H4	109.6	H11A—C11—H11B	109.5
N1—C5—C4	105.68 (8)	C8—C11—H11C	109.5
N1—C5—H5B	110.6	H11A—C11—H11C	109.5
C4—C5—H5B	110.6	H11B—C11—H11C	109.5
N1—C5—H5A	110.6	C7—N1—C1	121.03 (8)
C4—C5—H5A	110.6	C7—N1—C5	123.69 (8)
H5B—C5—H5A	108.7	C1—N1—C5	115.13 (8)
O1—C6—O2	120.96 (9)	C6—O2—C4	113.01 (7)
O1—C6—C1	126.91 (9)	C7—O4—C8	120.79 (7)
O2—C6—C1	112.10 (8)		
			
N1—C1—C2—C3	−56.55 (10)	C6—C1—N1—C7	123.18 (10)
C6—C1—C2—C3	58.77 (10)	C2—C1—N1—C7	−121.84 (9)
C1—C2—C3—C4	−1.58 (11)	C6—C1—N1—C5	−52.53 (11)
C2—C3—C4—O2	−57.69 (10)	C2—C1—N1—C5	62.46 (10)
C2—C3—C4—C5	60.76 (11)	C4—C5—N1—C7	−179.64 (9)
O2—C4—C5—N1	61.20 (10)	C4—C5—N1—C1	−4.06 (11)
C3—C4—C5—N1	−57.81 (10)	O1—C6—O2—C4	−177.27 (9)
N1—C1—C6—O1	−126.60 (11)	C1—C6—O2—C4	0.91 (11)
C2—C1—C6—O1	116.29 (11)	C5—C4—O2—C6	−61.30 (10)
N1—C1—C6—O2	55.36 (10)	C3—C4—O2—C6	60.21 (10)
C2—C1—C6—O2	−61.75 (10)	O3—C7—O4—C8	−4.98 (15)
O3—C7—N1—C1	5.74 (15)	N1—C7—O4—C8	175.60 (8)
O4—C7—N1—C1	−174.83 (8)	C10—C8—O4—C7	67.57 (11)
O3—C7—N1—C5	−178.93 (9)	C9—C8—O4—C7	−57.30 (12)
O4—C7—N1—C5	0.50 (13)	C11—C8—O4—C7	−175.35 (9)
