# Supplementary figures and images for: Crystal structure of (1R,4R)-tert-butyl 3-oxo-2-oxa-5-aza­bicyclo­[2.2.2]octane-5-carboxyl­ate
Source: Acta Crystallogr E Crystallogr Commun. 2015 Jun 6;71(Pt 7):o449–50. doi: 10.1107/S2056989015010476 (PMC4518923; doi:10.1107/S2056989015010476)

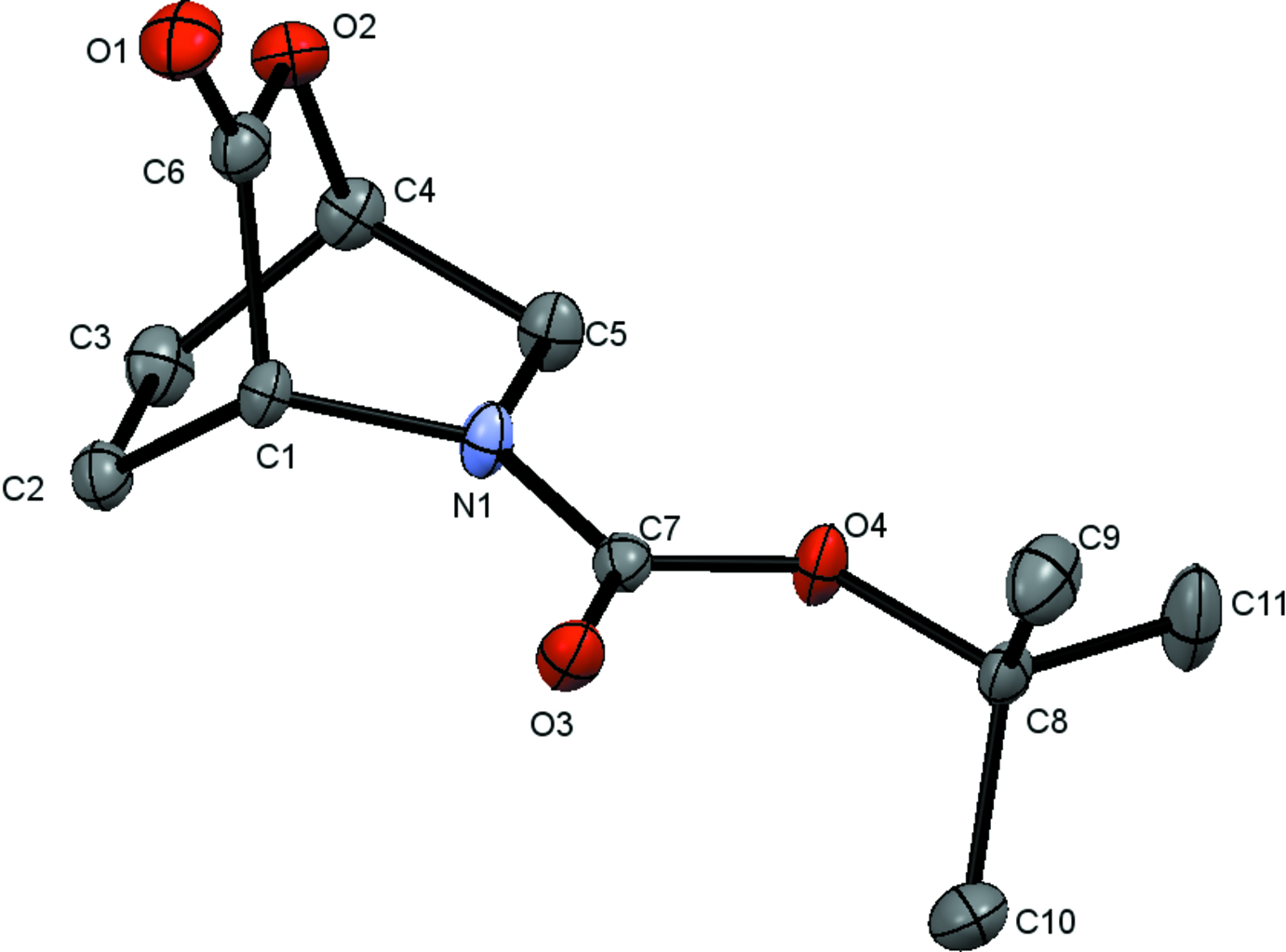

Supplement: Supplementary file 4 [file e-71-0o449-fig1.tif]

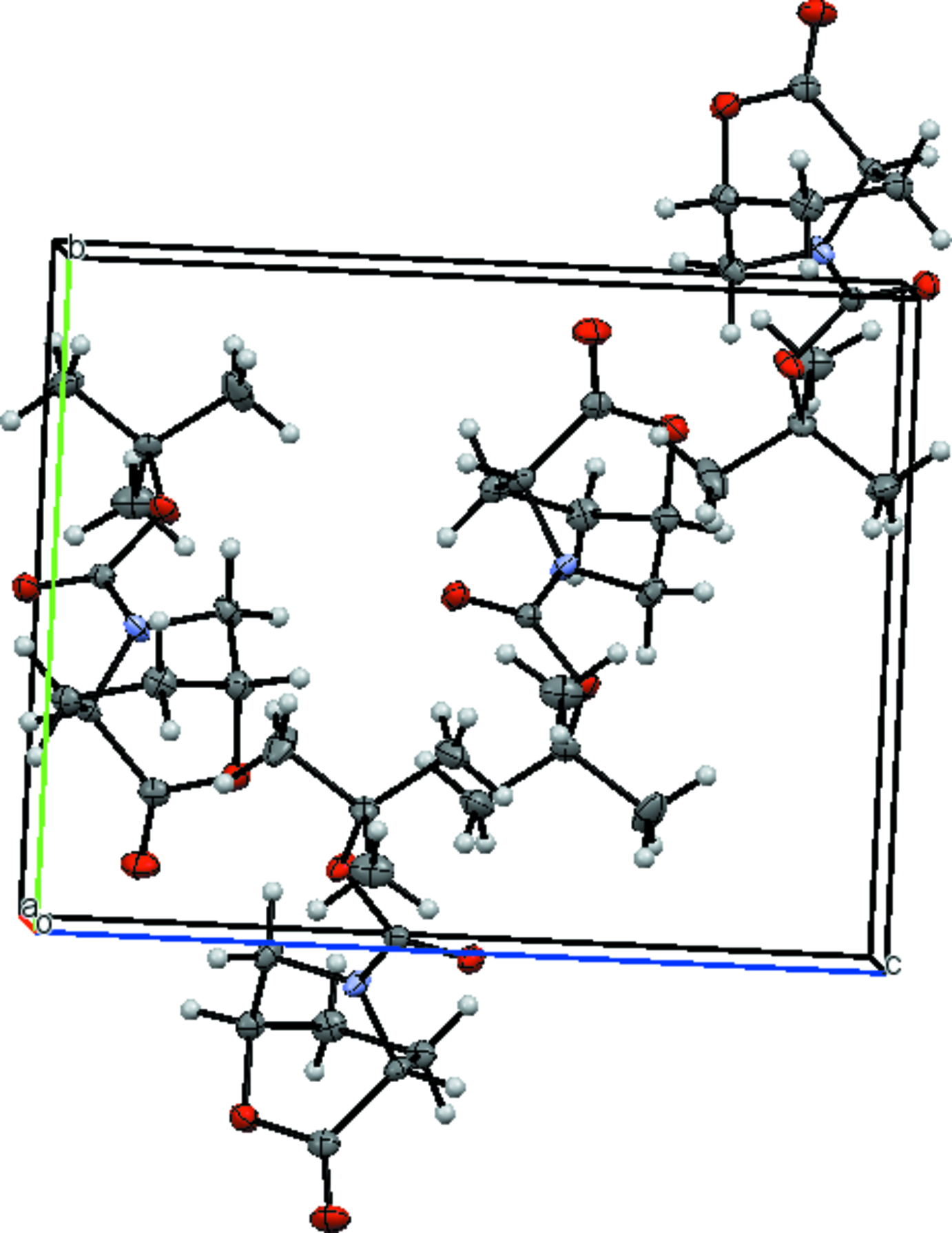

Supplement: Supplementary file 5 [file e-71-0o449-fig2.tif]
